# Accelerating Brain Imaging Using a Silent Spatial Encoding Axis

**DOI:** 10.1002/mrm.29350

**Published:** 2022-06-13

**Authors:** Edwin Versteeg, Dennis W. J. Klomp, Jeroen C. W. Siero

**Affiliations:** ^1^ Department of Radiology University Medical Center Utrecht Utrecht The Netherlands; ^2^ Spinoza Center for Neuroimaging Amsterdam Netherlands

**Keywords:** acceleration, gradient coil, gradient insert, magnetic resonance imaging, parallel imaging, quiet, silent

## Abstract

**Purpose:**

To characterize the acceleration capabilities of a silent head insert gradient axis that operates at the inaudible frequency of 20 kHz and a maximum gradient amplitude of 40 mT/m without inducing peripheral nerve stimulation.

**Methods:**

The silent gradient axis' acquisitions feature an oscillating gradient in the phase‐encoding direction that is played out on top of a cartesian readout, similarly as done in Wave‐CAIPI. The additional spatial encoding fills k‐space in readout lanes allowing for the acquisition of fewer phase‐encoding steps without increasing aliasing artifacts. Fully sampled 2D gradient echo datasets were acquired both with and without the silent readout. All scans were retrospectively undersampled (acceleration factors R = 1 to 12) to compare conventional SENSE acceleration and acceleration using the silent gradient. The silent gradient amplitude and the readout bandwidth were varied to investigate the effect on artifacts and g‐factor.

**Results:**

The silent readout reduced the g‐factor for all acceleration factors when compared to SENSE acceleration. Increasing the silent gradient amplitude from 31.5 mT/m to 40 mT/m at an acceleration factor of 10 yielded a reduction in the average g‐factor (g_avg_) from 1.3 ± 0.14 (g_max_ = 1.9) to 1.1 ± 0.09 (g_max_ = 1.6)_._ Furthermore, reducing the number of cycles increased the readout bandwidth and the g‐factor that reached g_avg_ = 1.5 ± 0.16 for a readout bandwidth of 651 Hz/pixel and an acceleration factor of R = 8.

**Conclusion:**

A silent gradient axis enables high acceleration factors up to R = 10 while maintaining a g‐factor close to unity (g_avg_ = 1.1 and g_max_ = 1.6) and can be acquired with clinically relevant readout bandwidths.

## Abstract 2

Click here for author‐reader discussions

## 1 INTRODUCTION

Over the past decades, parallel imaging techniques have become an essential part of MR‐imaging protocols, enabling shorter scan times and higher spatial resolutions for both research and clinical settings. The first parallel imaging techniques operated through regular undersampling of k‐space, which reduces scan time while introducing structural aliasing artifacts in the images. Removal of this aliasing uses the spatial sensitivities of the multi‐channel receive coil array in the reconstruction process. This can retrieve missing k‐space lines through interpolation kernels (SMASH/GRAPPA)^1^, ^2^ or unfold the aliased image in image‐space (SENSE).^3^ Generally, the maximum achievable undersampling in parallel imaging is limited by two main factors; the intrinsic loss in SNR from acquiring fewer k‐space lines and an additional noise‐amplification penalty, the so‐called g‐factor, determined by the spatial distribution of the aliasing artifacts and configuration of the receive coil array.

State‐of‐the‐art parallel imaging techniques aim to minimize the g‐factor by manipulating the spatial distribution of aliasing artifacts to optimally use the spatial sensitivities of the receive coils. One way is to alter the k‐space undersampling pattern for different phase (or slice) encoding steps. This can be done in a structured pattern (resembling a crystallographic grid) that shifts the aliasing artifacts to the edges of image‐space (like in CAIPIRINHA) or by introducing a pseudo‐random k‐space sampling that yields noise‐like aliasing artifacts (like in compressed sensing).^4^, ^5^ Alternatively, the g‐factor can be lowered by changing the spatial encoding by using additional rapidly oscillating gradients in one or more directions during the readout. This causes the aliasing artifacts to spread out in the readout direction and is the basis of methods like bunched‐phase encoding (BPE), FRONSAC, spread spectrum imaging, and Wave‐CAIPI.^6^, ^7^, ^8^, ^9^, ^10^ Here, the gradient amplitude of the oscillating gradient is the main parameter influencing the g‐factor and achievable acceleration factors.^11^ Importantly, this amplitude is limited by the biophysical effect of peripheral nerve stimulation (PNS) restricting the maximum slew rate.^12^ Moreover, introducing an extra switching gradient in the sequence leads to increased acoustic noise levels, which has to be limited to prevent hearing damage and might introduce patient anxiety.^13^, ^14^

This work explores the acceleration capabilities of a silent gradient axis.^15^ This silent gradient axis produces an inaudible oscillating gradient at 20 kHz without inducing PNS for gradient amplitudes up to at least 40 mT/m. Conventional MRI sound reduction methods reduce sound by slowly switching gradients resulting in a longer TR and scan time.^16^, ^17^ The silent gradient axis was developed to be combined with conventional MRI sound reduction methods and reduce sound without increasing scan time. This is achieved by providing extra spatial encoding that can accelerate scans and compensate for the longer TR of conventional MR sound reduction methods. For acceleration, the additional silent oscillating gradient induces a spread of aliasing in the readout direction in image space while in k‐space the oscillating gradient yields a denser sampling. This is similar to BPE and wave‐CAIPI albeit without an additional sound burden and with an order of magnitude higher slew rate. We expect this feature of the silent gradient can further increase the acceleration performance of parallel imaging compared to standard parallel imaging strategies such as SENSE. We explore this acceleration performance through retrospective undersampling of 2D GRE imaging data and by investigating the effect of silent gradient amplitude and readout bandwidth on the g‐factor.

## 2 METHODS

### 2.1 Silent gradient axis

The silent gradient axis approach consists of a head gradient insert and an audio amplifier. In this work, we used the same setup as in.^15^ Here, the gradient insert was designed for fast switching and ease‐of‐operation (Futura, Heerhugowaard, The Netherlands) and features a high efficiency (0.32 mT/m/A), low inductance (110 μH), and low weight (45 kg).^18^ The coil operates in the z‐direction (along the direction of the main magnetic field) and produces a gradient amplitude of 40 mT/m at 20 kHz when combined with the audio amplifier. To achieve this gradient amplitude, the gradient insert was made resonant at 20.34 kHz by adding tuning and matching capacitors, which maximized the obtainable gradient amplitude from the 18‐kW peak power and 450 V peak voltage available from the audio amplifier.

**FIGURE 1:**
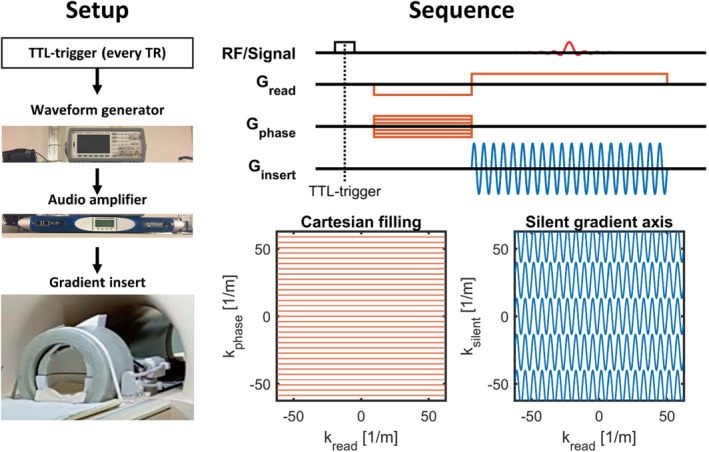
Schematic of the hardware setup and imaging sequence used for the silent gradient axis. Left, The hardware setup which is controlled by a waveform generator producing the sine‐waves and triggered each TR by a TTL‐pulse. Right, The imaging sequence used with the silent gradient axis. Here, the oscillating silent readout (blue) is played out simultaneously with the cartesian readout gradient (red), and operates in the phase‐encoding direction. This means that for each TR, a k‐space lane instead of a line is filled by the silent gradient axis

The silent gradient axis was controlled via a dedicated waveform generator that received gradient parameters (strength/frequency/starting time/number of cycles) from the scanner software. Here, the communication between the scanner software and waveform generator was handled by a custom python script (Python Software Foundation, https://www.python.org/) that used a SCPI (Standard Commands for Programmable Instruments) protocol over a LAN connection to send the parameters to the waveform generator. During imaging, a TTL‐trigger pulse was used to enable waveform generation each TR. A schematic overview of the silent gradient axis is shown in Figure 1.

### 2.2 Sequence and spatial encoding

The silent gradient axis produces an extra oscillating gradient in the phase or slice‐encoding direction on top of conventional cartesian phase encoding and readout, which are produced by the whole‐body gradients of the MR‐system. The silent gradient provides additional spatial encoding and yields a larger k‐space coverage per TR by filling k‐space with readout lanes instead of lines; hence, we dub the resulting readout as a “silent readout.” The sequence diagram and k‐space filling for the segmented readout with the silent gradient axis are shown in Figure 1.

Importantly, the readout lane width is one of the main parameters that influences the achievable acceleration using the silent gradient, as it determines the number of phase‐encoding steps that can be skipped while still acquiring a full k‐space. The readout lane width (Δk_silent_) depends on the gradient amplitude and frequency of the silent gradient and is given by the following equation:

(1) $$∆k_{\text{silent}}=\frac{\left(\gamma /2\pi \right)G}{\pi f}\;.$$

**FIGURE 2:**
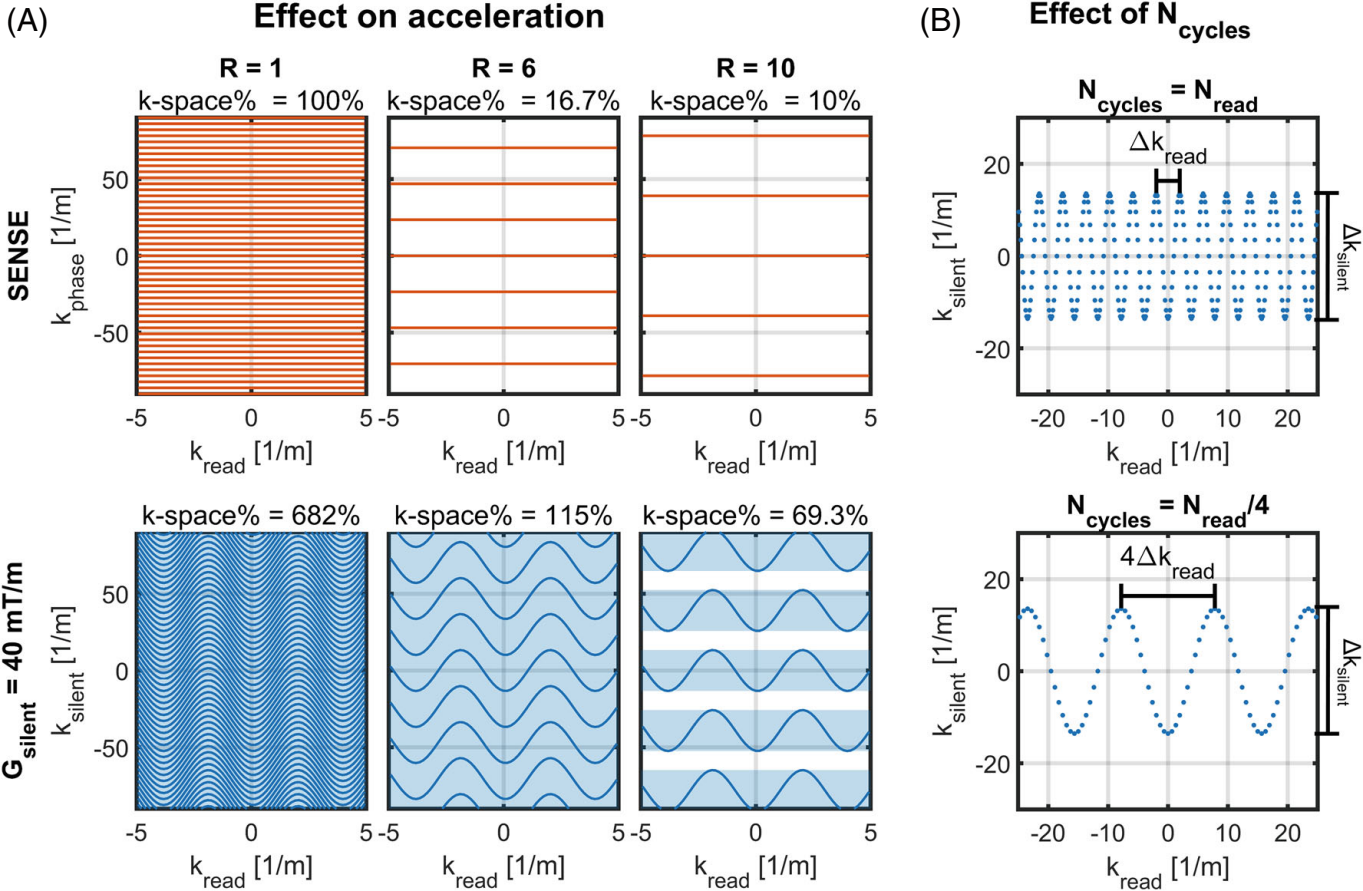
A, Schematic of the k‐space filling with and without the silent readout for different acceleration factors. Note that the extra encoding from the silent gradient axis even results in a high k‐space filling for an acceleration factor of R = 10. B, Schematic of the effect of the number of cycles played out during the readout on the k‐space sampling. Here, the use of fewer cycles (bottom) results in a larger average distance between samples in the readout direction (k_read_), which means that the scan will effectively be undersampled in the readout direction

In Equation (1), Δk_silent_ is the readout lane width in k‐space, γ is the gyromagnetic ratio in rad/s/T, G is the oscillating silent gradient amplitude in T/m, and f is the silent gradient oscillation frequency in Hz. Equation (1) can be used to determine gain in imaging efficiency for the silent gradient axis. For example, a conventional cartesian acquisition has phase‐encoding steps of Δk = 3.9 m^−1^ for a FOV of 256 mm. In comparison, the silent gradient driven at 40 mT/m and 20 kHz yields a readout lane width of Δk_silent_ = 27.1 m^−1^. Consequently, the silent gradient can use Δk_silent_/Δk = 6.9‐fold larger phase‐encoding steps per TR to fill k‐space, resulting in a corresponding reduction in scan time. Figure 2A shows the effect of the readout lanes on k‐space filling for different acceleration factors. Here, the k‐space filling was defined as follows; for scans without silent readout, the k‐space filling was calculated as the fraction of k‐space lines acquired compared to a fully sampled k‐space. For scans with a silent readout, the k‐space filling was calculated as the fraction of the total readout lane width (Δk_silent_ × N_lanes‐sampled_) compared to the width (k_max_) of a fully sampled k‐space. Note, this can yield a k‐space filling larger than 100% as seen in (Figure 2A).

The achievable acceleration is also influenced by the number of sine cycles played out during the readout, as this determines the k‐space sampling density in each readout lane. Here, the use of more cycles results in a higher k‐space sampling density by reducing the average distance between k‐space samples, which relates to fewer aliasing artifacts. A schematic depiction of the effect of the number of cycles on the k‐space sampling density is shown in Figure 2B. Additionally, the fixed oscillation frequency of the silent gradient means that the number of cycles directly determines the duration of the acquisition window, the readout bandwidth, and the strength of the conventional (cartesian) readout gradient. Here, the relationship between number of cycles and readout gradient strength is given by:

(2) $$G_{\text{read}}=\frac{f}{N_{\text{cycles}}\Delta x_{\text{read}}\left(\gamma /2\pi \right)}\;.$$

In Equation (2), G_read_ is the readout gradient strength in T/m, f is the oscillation frequency of the silent gradient in Hz, N_cycles_ is the number of cycles played out during the readout, Δx_read_ is the voxel size in the direction of the readout gradient in meters, and γ is the gyromagnetic ratio in rad/s/T. Importantly, Equation (2) also directly relates the number of cycles to SNR, as SNR can be seen as inversely proportional to the square root of the readout bandwidth (BW), which linearly depends on the readout gradient strength.

### 2.3 Imaging experiments

The silent gradient was positioned in a 7T MR‐scanner (Achieva, Philips, Best, The Netherlands). Here, the silent gradient was positioned such that the isocenter of the silent gradient lined up with the isocenter of the whole‐body gradients. A birdcage RF‐coil integrated in the gradient insert was used for transmit and a 32‐channel receive coil (Nova Medical, Wilmington, MA, USA) was positioned in the gradient insert for receive.^18^ For all experiments, the silent gradient was operated at a frequency of 20.34 kHz. The acceleration performance was investigated by retrospective undersampling of a fully sampled 2D gradient echo sequence featuring the silent readout while changing (1) the readout lane width by changing the silent gradient amplitude and (2) the number of cycles by changing the readout bandwidth.

1. The readout lane width was varied by driving the silent gradient at 31.5 mT/m and 40 mT/m, which yielded readout lane widths of 21.0 m^−1^ and 26.6 m^−1^, respectively. The following sequence parameters were used for the 2D gradient echo sequence: in‐plane resolution = 1x1 mm^2^, slice‐thickness = 2 mm, FOV= 256 × 256 mm^2^, flip angle = 22°, readout bandwidth = 81 Hz/pixel (G_read_ = 1.9 mT/m), 24‐fold oversampling in the readout direction (f_sampling_ = 488 kHz, the actual sampling frequency during the acquisition window), startup‐echoes = 50, number of cycles during readout = 256, TR = 62 ms and TE = 11 ms. Additionally, a scan without the silent readout but with identical sequence parameters was acquired to compare with SENSE undersampling. The high oversampling factor was necessary due to the large voxel spreading caused by the silent gradient. An example of this can be seen in Supporting Information Figure S1, which is available online.
2. The effect of the number of cycles during readout was explored using five scans with the number of cycles during the readout varying from 32 to 256 (specifically: 32, 42⅔, 64, 128, 256), which resulted in the readout bandwidth varying from 650 Hz/pixel to 81 Hz/pixel. This range of readout bandwidths corresponded to a readout gradient G_read_ that varied from 15.3 mT/m to 1.9 mT/m. Furthermore, the maximum achievable sample rate (limited by scanner data rate) was used in all scans, which was f_sampling_ = 488 kHz for 256 cycles and f_sampling_ = 976 kHz for all other scans. All scans were retrospectively undersampled six‐fold and featured a slice thickness of 3 mm to ensure sufficient SNR. All other scan parameters were identical to the aforementioned gradient echo sequence.

Informed consent was given by all volunteers in accordance with the local Institutional Review Board for all aforementioned scans.

### 2.4 Retrospective undersampling and image reconstruction

Retrospective undersampling and image reconstruction were performed offline in MATLAB (Mathworks, Natick, MA, USA). A maximum acceleration factor of R = 12 was used to ensure sufficient SNR for the image reconstruction. The retrospective undersampling patterns were centered around k = 0 to mimic the sampling of a prospectively undersampled scan. The k‐space filling of each undersampling pattern was calculated to investigate its effect on acceleration performance.

The scans were reconstructed with an identical image reconstruction pipeline, which used a generalized conjugate gradient (CG) SENSE algorithm to perform iterative reconstruction.^19^ The raw data, k‐space trajectory and coil sensitivities were provided as an input to the CG‐SENSE reconstruction, and a non‐uniform Fourier transform (GPUNUFFT^20^) was used for all reconstructions. K‐space trajectories for the cartesian readout were based on the ideal gradient waveforms played out by the scanner while the silent readout was generated using timing and amplitude information obtained from field camera measurements (Dynamic Field Camera, Skope, Switzerland). The field camera measurements were obtained using 16 field probes that were positioned around the isocenter of the silent gradient. Furthermore, density compensation was applied to acquired data using the silent readout to compensate for oscillations in k‐space sample density. Coil sensitivities were obtained from a separate low‐resolution but similar cartesian GRE scan and re‐gridded to the high‐resolution target images. G‐factor maps were obtained by using the pseudo‐multiple replica method with 100 replicas.^21^ Average (g_avg_) and maximum (g_max_) g‐factors were calculated from the g‐factor maps. For the SENSE undersampled scans, the g‐factor calculations were only possible for acceleration factors lower than R = 8, as aliasing artifacts prevented accurate calculations for higher acceleration factors. RMS error (RMSE) was calculated to quantify the artifact level with respect to the fully sampled images.

#### 2.4.1 Simulations

To accompany the in‐vivo experiments in this work, we also performed simulations on a digital phantom. Here, we investigated a wider range of silent gradient amplitudes (0–70 mT/m) and readout bandwidths (81–1270 Hz/pix) and quantified the acceleration performance in terms of g‐factor for acceleration factors R = 5, 8, 10, and 12. The simulations allowed us to verify the trends in the g‐factor observed in the in‐vivo experimental data and featured the same reconstruction pipeline. The simulations can be found in the Supporting Information Figure S2–S5, “G‐factor simulations on a digital phantom”.

## 3 RESULTS

### 3.1 Retrospective undersampling

**FIGURE 3:**
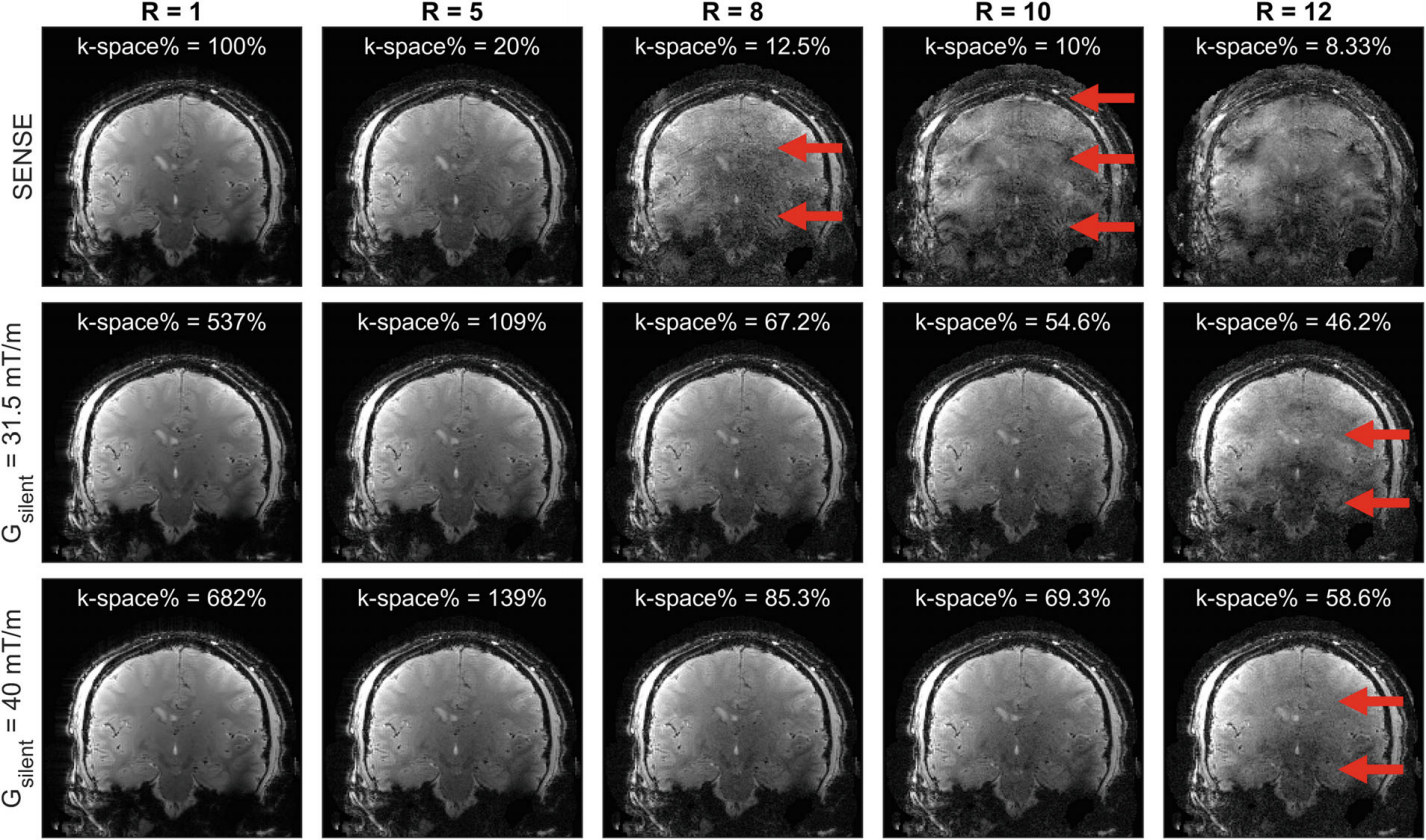
Results of the retrospective undersampling of 2D imaging data (FOV 256 × 256 mm^2^, voxel size = 1 × 1 × 2 mm^3^) and the k‐space filling for each acceleration factor R. Top row: SENSE reconstruction of the acquisition without silent gradient for a range of acceleration (SENSE) factors. Note that significant aliasing artifacts were present for higher acceleration factors (R > 5). Middle row: Reconstructions for the scans with a silent gradient amplitude of 31.5 mT/m. Note that aliasing artifacts started to appear at an acceleration factor of R > 10. Bottom row: Reconstructions for the scans with a silent gradient amplitude of 40 mT/m. Note that the higher amplitude resulted in a higher k‐space coverage that reduced aliasing artifacts at R = 12

Figure 3 shows the reconstruction results for various acceleration factors and readout lane widths. In the SENSE undersampling, aliasing artifacts and increased noise were observed for acceleration factors higher than R = 5. The aliasing artifacts and noise were substantially reduced when using the silent readout at a gradient amplitude of 31.5 mT/m, which visually started showing aliasing artifacts at R = 12. Increasing the silent gradient amplitude to 40 mT/m resulted in an additional reduction in noise and aliasing artifacts, especially noticeable for an acceleration factor of R = 12. Generally, the decrease in aliasing artifacts and noise when using the silent readout could be attributed to the increased filling of k‐space, which was approximately five to eight times higher for the silent gradient when compared to SENSE undersampling.

**FIGURE 4:**
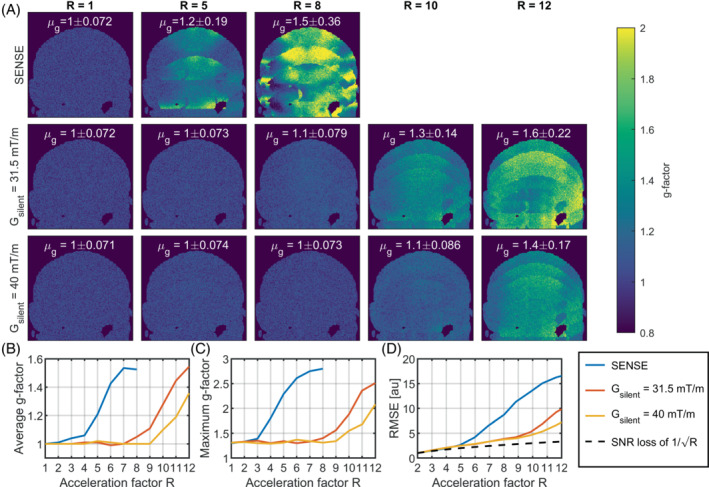
A, G‐factor maps for the reconstructions Figure 2. Top row: G‐factors for the SENSE reconstructions Middle row: G‐factors for the silent gradient at 31.5 mT/m. Here, the g‐factor stayed close to unity for undersampling factors up to R = 8. Bottom row: G‐factors for the silent gradient at 40 mT/m. Note that compared to the amplitude of 31.5 mT/m, the higher gradient amplitude lowered the g‐factor for all acceleration factors. B, The spatial average g‐factor for the different reconstructions. Note that the scans with silent gradient featured an average g‐factor close to unity for acceleration factors up to R = 10. C, The maximum g‐factor for the different reconstructions. D, The RMSE of the accelerated image reconstructions compared to the non‐accelerated case (R = 1)

The resulting noise from the different scans and reconstructions is shown in Figure 4. Here, Figure 4A–C show g‐factor maps, average g‐factors, maximum g‐factors for the various acceleration factors and gradient amplitudes. The g‐factor maps of the SENSE undersampling showed a rapid increase of spatially varying noise amplification for increasing acceleration factors. Using the silent readout, the g‐factor maps showed average g‐factors close to unity up to an acceleration factor of R = 8 (g_avg_ = 1.1/g_max_ = 1.4) when using a gradient amplitude of 31.5 mT/m and up to R = 10 for an amplitude of 40 mT/m (g_avg_ = 1.1/g_max_ = 1.6). In both cases, the average g‐factors increased rapidly for higher acceleration factors R > 8. The reduction in g‐factor with silent gradient amplitude was also observed in simulated data as can be seen in Supporting Information Figure S2 in Appendix S1.

Figure 4D shows the effect of acceleration on the RMSE. Here, the SENSE undersampling started to deviate from the theoretical increase in RMSE with 1/√R at acceleration factors of R > 5, which matched with the increase in aliasing artifacts observed in Figure 3. Using the silent readout, the RMSE was lower than the SENSE undersampling acquisitions for all acceleration factors, which matched the decrease in aliasing artifacts observed in Figure 3 when using the silent gradient. Additionally, the RMSE was also reduced when increasing the silent gradient amplitude from 31.5 mT/m to 40 mT/m, which was observed as a decrease in aliasing artifacts in the R = 12 reconstruction in Figure 3.

### 3.2 Effect of number of cycles

**FIGURE 5:**
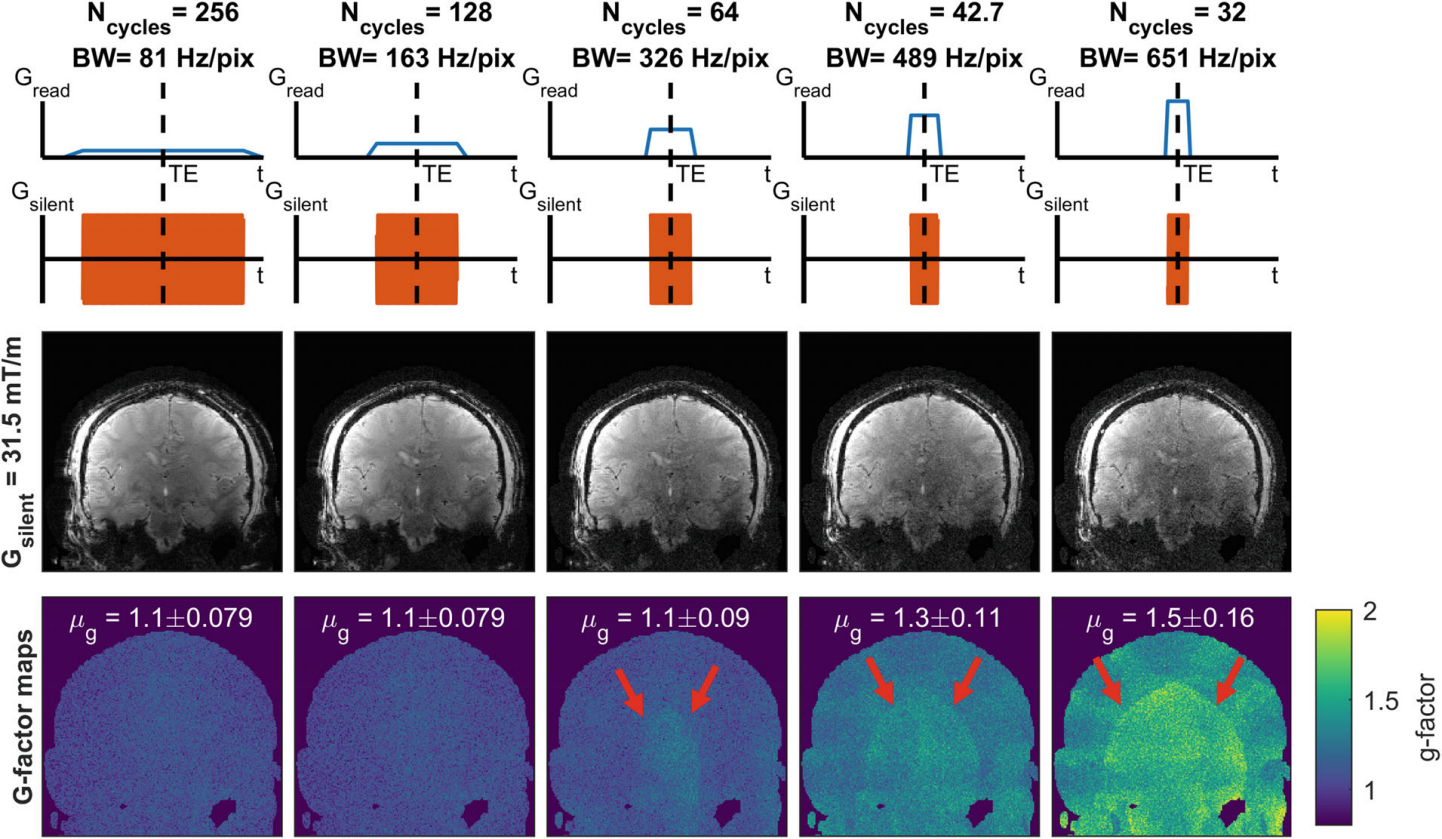
Results for the effect of number of cycles (readout duration/bandwidth) on the images acquired using the silent gradient for R = 8. Top row: schematic of the readout used for each reconstruction. Middle row: reconstruction for different N_cycles_ (readout durations/bandwidth). Note that shortening the readout duration reduced SNR (due to the higher readout bandwidth) but did not introduce any additional aliasing artifacts. Bottom row: g‐factor maps for the different readout directions. Note that a small increase in g‐factor was present for shorter readouts. These short readouts resulted in effective undersampling in the readout direction (the noise amplification pattern is in the right‐left direction) as on average the sample distance was larger than the Nyquist limit (see also Figure 2)

Figure 5A shows the effect of changing the number readout cycles played out during the readout on the image reconstruction and g‐factor for a fixed acceleration factor of R = 8. A decrease in the number of cycles did not yield any additional aliasing artifacts in the images but resulted in an SNR decrease due to the increased readout bandwidth from the shorter acquisition window (Figure 5B). Furthermore, an increase in g‐factor was observed for the case of N_cycles_ < 64, which could be attributed to an effective undersampling in the readout direction due to a low k‐space sampling density in that direction. A similar trend was observed in simulation data where a decrease in cycles resulted in an increase in g‐factor for different gradient amplitudes. This was visualized in Supporting Information Figure S3.

## 4 DISCUSSION

In this work, we have demonstrated the acceleration potential of an extra silent gradient axis, which enables high acceleration factors up to a factor of R = 10 with g‐factors close to unity without introducing additional audible sound. These high acceleration factors were possible due to the silent gradient's additional spatial encoding. Even at very high acceleration factors (R > 8), a large k‐space filling of more than 50% is reached limiting aliasing artifacts. The presented approach is similar to other encoding methods like BPE and wave‐CAIPI albeit at a 25‐fold higher slew rate resulting in a combination of gradient amplitude (40 mT/m) and frequency (20 kHz) that has not been achieved before.^6^, ^10^

The maximum amplitude of the silent gradient of 40 mT/m used in this work was comparable to the maximum gradient amplitude of whole‐body gradient systems. However, the slew rate needed to achieve this was 5112 T/m/s, which is ∼25 fold higher when compared to the 200 T/m/s slew rate limit of conventional whole‐body gradients. To put this into perspective, other high performance head gradients can reach slew rates of 500–1200 T/m/s, which would mean a maximum gradient amplitude of 4–9.5 mT/m at 20 kHz.^22^, ^23^, ^24^

Compared to conventional SENSE undersampling, the extra silent readout approach was found to substantially decrease g‐factor noise and aliasing artifacts. Currently, the gradient amplitude was limited to 40 mT/m by the maximum power output of the audio amplifier. However, a lower g‐factor at higher acceleration factors R > 10 might be feasible with an even higher silent gradient amplitude as currently substantial PNS has not been reported. Moreover, the image quality of such an approach will ultimately be limited by the intrinsic SNR decrease with undersampling.

The current design of the silent gradient axis limits the gradient induced acceleration to the z‐direction. For 2D imaging, this means only coronal or sagittal scan orientations can be acquired with this setup. However, such a limitation is not present for 3D imaging as the silent readout can be applied in either the phase and slice encoding direction. Additionally, the design of the silent gradient axis could be extended to a different orientation (X or Y) or multiple axes to allow for transverse scan orientations in 2D imaging and potentially even higher acceleration factors.^25^ In this work, 2D imaging was used to simplify the computation of retrospective undersampling patterns and g‐factors. However, there are no intrinsic limitations to extend the method to 3D or combine it with other undersampling strategies like CAIPIRINHA and compressed sensing,^4^, ^5^ which might also benefit the g‐factor at high acceleration factors.

### 4.1 Non‐silent acceleration potential

We have also shown that the silent readout can be combined with a higher readout bandwidth to enable acceleration. Here, shortening the readout was found to introduce an effective undersampling in the readout direction, which yielded an increase of 20% in g‐factor for a short readout (1.5 ms) with 32 cycles. Importantly, the range of readout bandwidths tested in this work were similar to bandwidths used in anatomical imaging sequences like FLAIR and MPRAGE, which would thus be suitable for use with the silent readout with only minimal modifications.^26^

## 5 CONCLUSIONS

All in all, we have shown that a silent gradient axis can be used to achieve high acceleration factors up to R = 10 while maintaining a g‐factor close to unity for 2D imaging and can be acquired with clinically relevant readout bandwidths. Importantly, this is achieved without introducing any additional audible sound into the MR‐system nor any substantial sensation of PNS. As such, the silent gradient axis is expected to have a wide range of both clinical and research applications in which it can be used to increase imaging speed while maintaining patient comfort.

## Supporting information


**Appendix S1** Supplementary Information
