# Supplementary material for: Accelerating Brain Imaging Using a Silent Spatial Encoding Axis
Source: Magn Reson Med. 2022 Jun 13;88(4):1785–93. doi: 10.1002/mrm.29350 (PMC9544176; doi:10.1002/mrm.29350)
Supplement: Supplementary file 1 — Appendix S1 Supplementary Information [file MRM-88-1785-s001.docx]

**Supporting information: Voxel spreading**

Figure S1 Voxel spreading effect for the silent gradient. Top: Voxel spreading in image domain for G_silent_ = 40 mT/m with an oversampling factor of 24. Bottom: Collapsed voxel spreading, which was obtained by summing over the receive channels and in the direction of the silent gradient. Note the decrease in signal towards the edges of the oversampled field-of-view which originates from the roll-off of the ADC filter.

**Supporting information: G-factor simulations on a digital phantom:**

**Methods**

G-factor simulations were performed on a simulated Shepp-Logan phantom using the same imaging parameters as the experimental data i.e.: a field-of-view of 256 x 256 mm^2^ and an in-plane voxel size of 1 x 1 mm^2^. Coil sensitivities were generated for a 32-channel receive array consisting of 4 rows (in the z-direction) of 8 receive coils (at a radius of 10 cm) which have similar characteristics as the receive array used for the experimental data. Each receive coil was rectangular with sides of 6 cm. The complex coil sensitivities were calculated using Biot-Savart’s law. The phantom and receive array setup are shown in Figure S2.


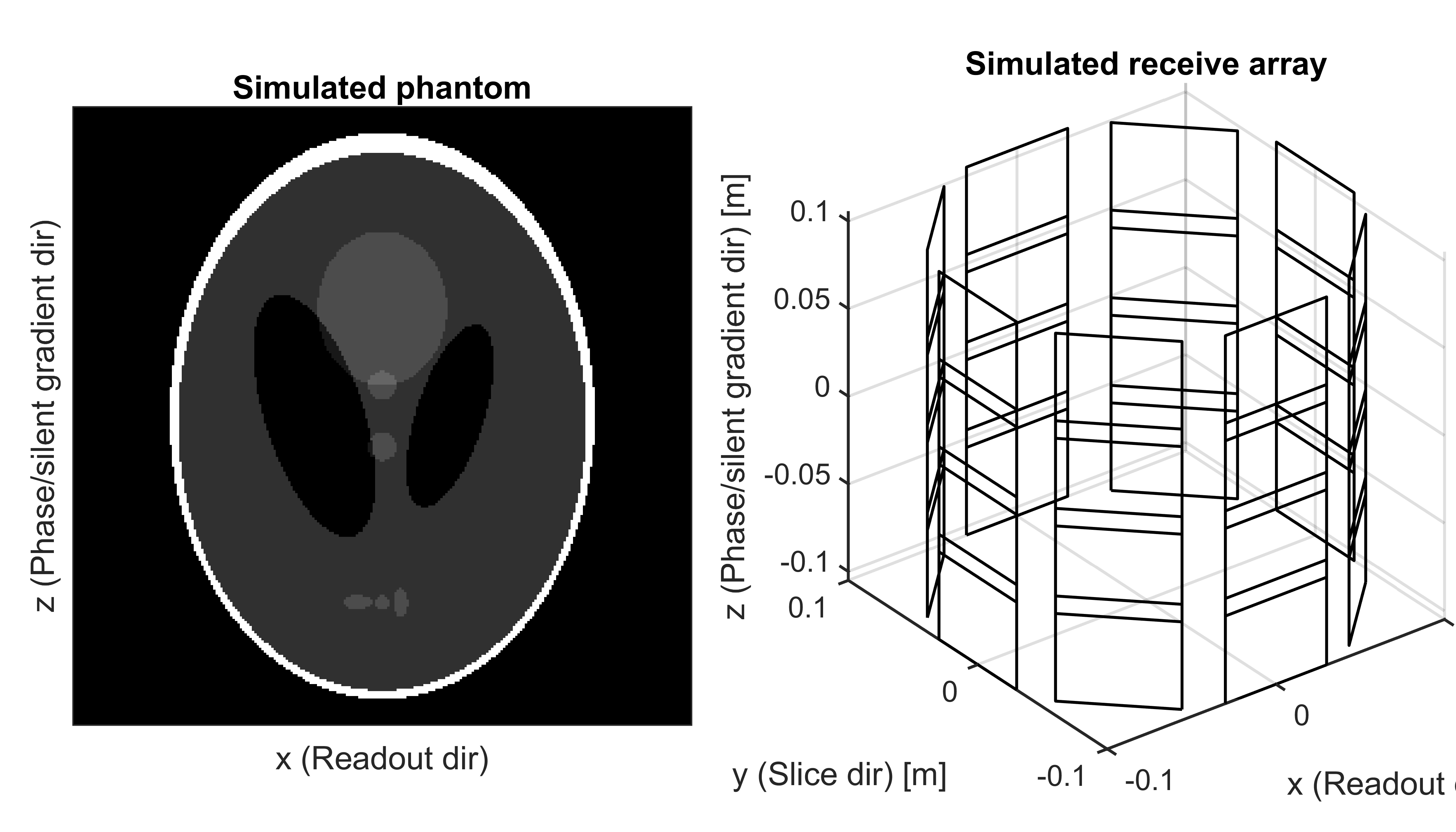


Figure S2 Left: the simulated Shepp-Logan phantom. Right: the simulated 32-channel receive array

Similar to the experimental data in the manuscript, two sets of simulations were performed in which the acceleration performance was investigated by retrospective undersampling of a simulated fully-sampled 2D dataset featuring the silent readout while changing 1) the readout lane width by changing the silent gradient amplitude while keeping the readout bandwidth constant (at 81 Hz/pixel) 2) the number of cycles by increasing the readout bandwidth. Here, the silent gradient amplitude was varied between G_silent_ = 0 mT/m and 70 mT/m in steps of 5 mT/m, while the number of cycles was varied between 256 and 16 (readout bandwidth between 81 Hz/pix and 1270 Hz/pix corresponding to a readout gradient ranging from 1.9 mT/m to 29.8 mT/m). For case 1), the simulations were performed for the same acceleration factors as in Figure 3 of the manuscript (R = 1/5/8/10/12). In case 2), the simulations were performed for the acceleration factors R = 5 and 8.

Fully-sampled k-spaces were generated for each combination of silent gradient amplitude and readout bandwidth using a non-uniform Fourier transform (NUFFT). The fully-sampled k-spaces were retrospectively undersampled and reconstructed using the same reconstruction pipeline as the experimental data. G-factor maps were calculated using the pseudo-replica method using 50 replicas for all the combinations of parameters in the simulations. The resulting g-factor maps were summarized by calculating the average and maximum g-factors.

**Results and discussion**


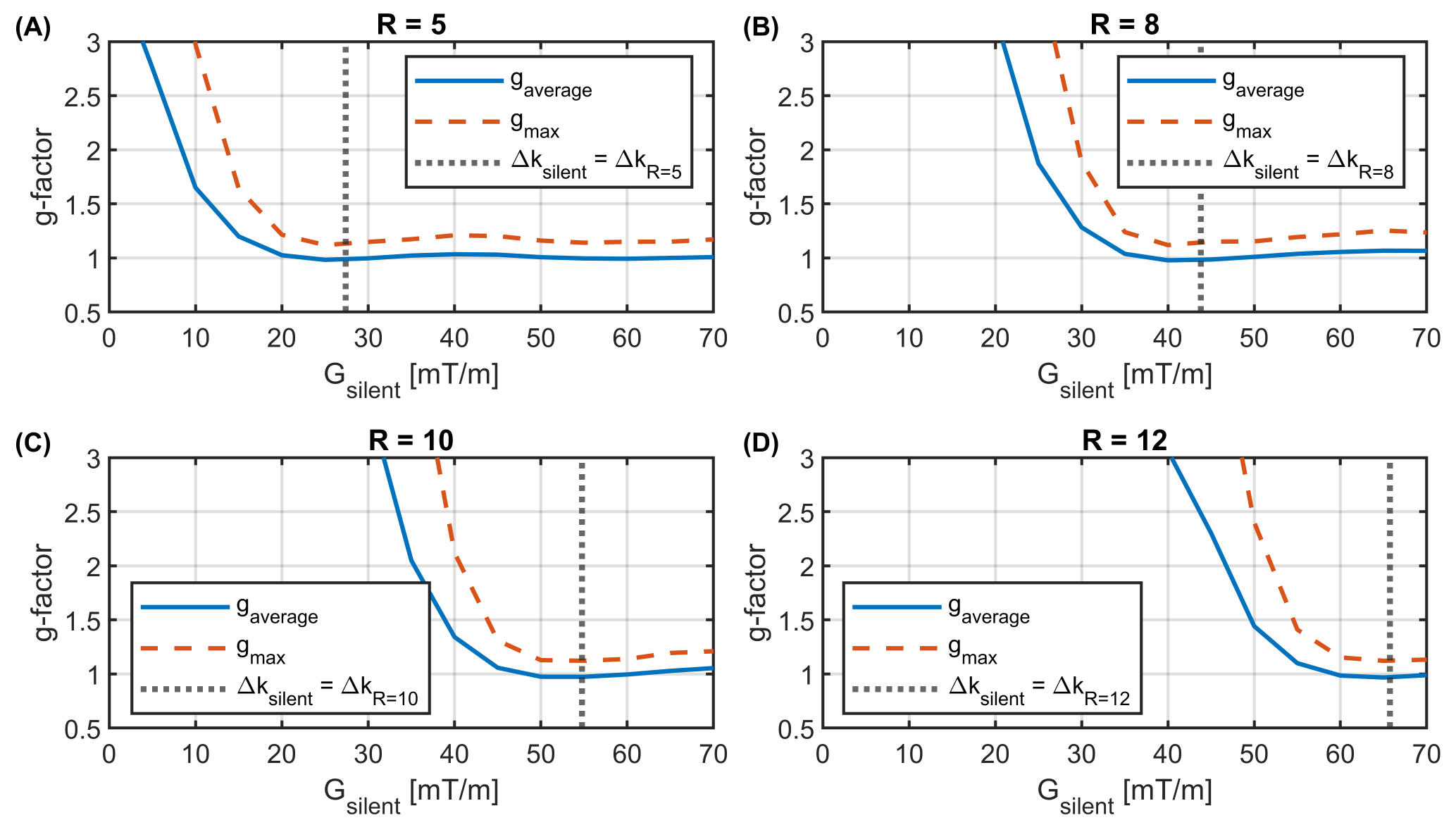


Figure S3: Results for the average and maximum g-factor for different acceleration factors and silent gradient amplitudes. Results are shown for an acceleration factor of R = 5 (A), R = 8 (B), R= 10 (C) and R = 12 (D), which are the same acceleration factors shown in Figure 3 and 4 of the manuscript. The dotted line in each subplot indicates the amplitude at which the lane width (Δk_silent_) equals the phase-encode steps used for the accelerated scan (Δk_R_=R/FOV).

Figure S3 shows the average and maximum g-factor for acceleration factors and a range of silent gradient amplitudes. Here, both the average and maximum g-factor decrease with increasing silent gradient amplitude for all acceleration factors. This is similar to the behavior found in Figures 3 and 4, where an increase in silent gradient amplitude from 31.5 to 40 mT/m was found to decrease the g-factor. The values for the average g-factor found in the simulations were higher than those found in experiment: e.g for an acceleration factor for R = 10 and G_silent_ = 40 mT/m an average g-factor of 1.3 was found in simulation while the experimental data showed a g-factor of 1.1. This difference in average g-factor is most likely due to the differences between the coil array used for the simulations and the actual coil array used in the experiments.


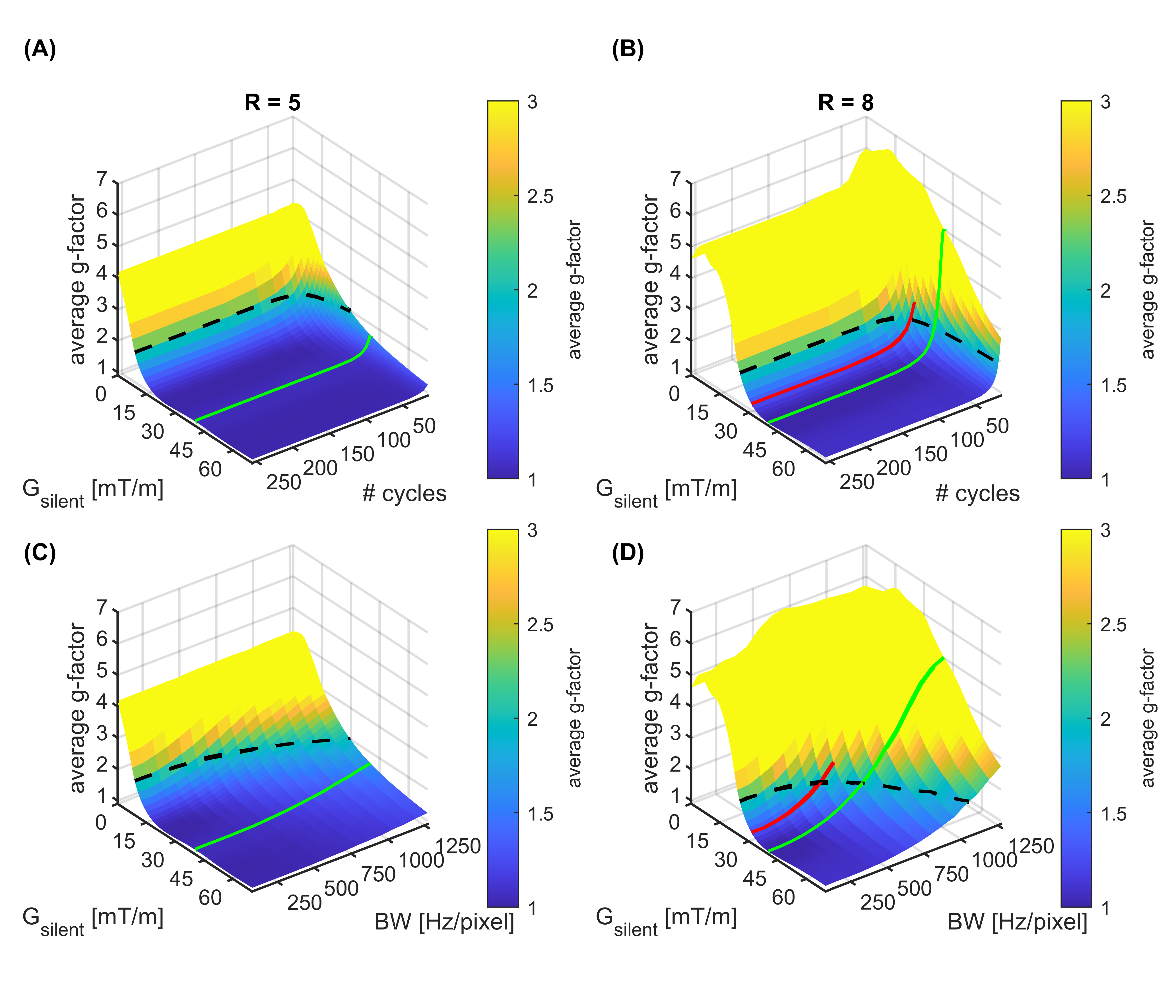


Figure S4 Simulation results for the effect of #cycles/readout bandwidth and silent gradient amplitude on the average g-factor. (A) Results for an acceleration factor R = 5 with #cycles vs silent gradient amplitude on the axes; (B) Results for an acceleration factor R = 5 with #cyles vs silent gradient amplitude on the axes. Here, the red line indicates the parameter combinations used in Figure 5 of the manuscript; (C) Results for an acceleration factor R = 5 with readout bandwidth vs silent gradient amplitude on the axes; (D) Results for an acceleration factor R = 8 with readout bandwidth vs silent gradient amplitude on the axes. Here, the red line indicates the parameter combinations used in Figure 5 of the manuscript. In all subplots, the green lines indicate the current hardware limit of 40 mT/m while the black dashed lines indicate the isocontour where the g-factor is equal to 2.

Figure S4 shows the simulation results for different combinations of readout bandwidth, acceleration factor and silent gradient amplitude. Here, Figure S4A/C show that with the current hardware limit of 40 mT/m a g-factor lower than 2 should be feasible for readout bandwidths up to 1270 Hz/pixel with an acceleration factor of R = 5. In Figure S4B/D, the higher acceleration factor of R = 8 yields a faster increase of g-factor with readout bandwidth, limiting the range of readout bandwidth for which a g-factor lower than 2 is achievable.
